# Supplementary material for: Epigenome-wide association study of household air pollution exposure in an area with high lung cancer incidence
Source: medRxiv. 2025 Apr 4:2025.04.03.25325041. Preprint. [Version 1] doi: 10.1101/2025.04.03.25325041 (PMC11998846; doi:10.1101/2025.04.03.25325041)

Supplemental Material

**Epigenome-wide association study of household air pollution exposure in an area with high lung cancer incidence.**

Mohammad L Rahman^1^, Lützen Portengen^2^, Batel Blechter^1^, Charles E. Breeze^1^, Jason Y.Y. Wong^3^, Wei Hu^1^, George S. Downward^2,4^, Yongliang Zhang^2^, Andres Cardenas^5^, Bou Ning^6^, Jihua Li^7^, Kaiyun Yang^8^, H. Dean Hosgood^9^, Debra T. Silverman^1^, Nathaniel Rothman^1^, Yunchao Huang^8#^, Roel Vermeulen^2#^, Qing Lan^1#^

**Figure S1**. Beta density plots of leukocyte DNA methylation in never-smoking

women. The x-axis shows the DNA methylation beta values, while the y-axis

indicates their density. Panel A shows the plot before data quality control, Panel B

after quality control, and Panel C after normalization using the beta mixture quantile

dilation (BMIQ) method.


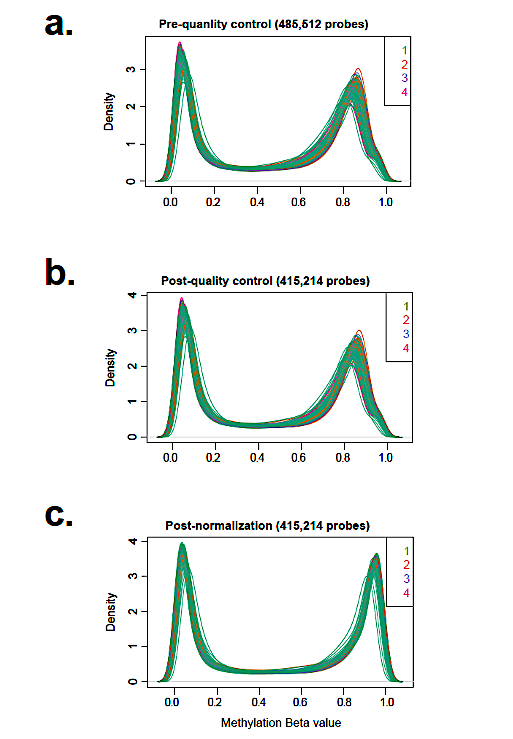


**Figure S2**. Pearson’s correlations between beta coefficients of PAH clusters (x-axis) and 5-

MC (y-axis) across respective exposure windows for (a) previously identified smoking-related

CpG sites (2,476 CpGs) and (b) top epigenome-wide significant sites (782 CpGs; FDR < 0.05).


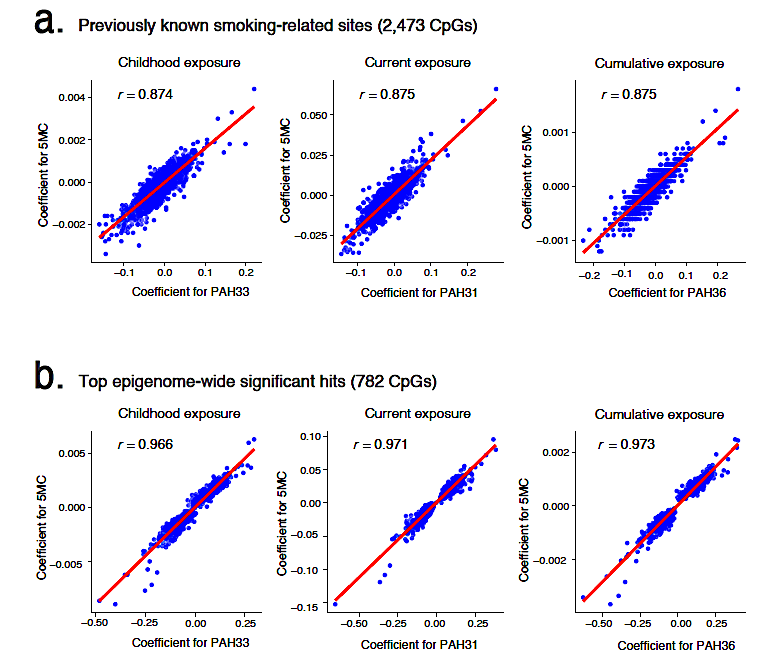

Supplement: Supplement 1 [file media-1.docx]
